# Optimizing Outcomes in Total Femur Replacement: Complications, Management Strategies, and Lessons Learned

**DOI:** 10.3390/medicina62050809

**Published:** 2026-04-24

**Authors:** Zofia Wrześniak, Bartłomiej Wilk, Łukasz Pulik, Grzegorz Guzik, Paweł Łęgosz

**Affiliations:** 1 Faculty of Medicine, Medical University of Warsaw, Żwirki i Wigury 61, 02-091 Warsaw, Poland; wilk.wilkbartlomiej@gmail.com; 2 Department of Orthopaedics and Traumatology, Medical University of Warsaw, ul. Lindleya 4, 02-005 Warsaw, Poland; lukasz.pulik@wum.edu.pl (Ł.P.); pawel.legosz@wum.edu.pl (P.Ł.); 3 Oncological Orthopaedics Department, Podkarpacki Regional Oncological Centre, ul. ks. Józefa Bielawskiego 18, 36-200 Brzozów, Poland; grzegorz.guzik@vp.pl

**Keywords:** total femur replacement, arthroplasty, endoprosthesis

## Abstract

*Background and Objectives*: Total femoral replacement (TFR) was originally developed for limb salvage following the resection of malignant tumors. Over time, its indications have expanded, now serving as a reconstructive option for failed endoprosthetic replacements and severe trauma cases. Despite its advantages, TFR is a highly complex surgical procedure associated with significant complication rates. This study aims to analyze the management of complications and propose strategies to mitigate associated risks. *Materials and Methods*: This is a retrospective study conducted on patients from two independent hospitals who underwent TFR for different reasons. *Results*: Nineteen patients were included: eight underwent TFR for oncological indications, while 11 had the procedure as a revision following failed endoprosthetic arthroplasty or trauma. Postoperative complications were observed in 10 patients (53%), including hip dislocation (21%), mechanical implant failure (11%), infection (21%), wound healing complications (26%), and metal allergy symptoms (5%). Revision surgery was required in six patients (32%), but no cases necessitated amputation. *Conclusions*: TFR is associated with a high risk of complications, with infection and wound healing issues being the most prevalent. In our experience effective complication management strategies should include early intervention, considering TFR at an earlier stage in non-oncological patients to minimize multiple revision surgeries; allergy screening, assessing for potential metal hypersensitivity preoperatively; dislocation prevention, implementing dual mobility bearings to reduce instability; infection control, utilizing intraoperative local antibiotic therapy in revision cases; and wound management, applying vacuum-assisted closure (VAC) therapy postoperatively to enhance wound healing. Implementing these strategies may improve patient outcomes and reduce the burden of complications associated with TFR.

## 1. Introduction

Total femoral replacement (TFR) was developed in 1965 as a complete replacement of the femur after resection of malignant neoplasms [1]. Throughout the years the indications for this procedure have evolved. Nowadays it is a recognized limb salvage procedure performed as an alternative to lower limb amputation after failed endoprosthetic replacements in the setting of massive bone loss. TFR is expected to be performed more frequently as the worldwide rate of revision arthroplasty increases due to the increased arthroplasty surgical volume and survival rates of patients and their underlying diseases, exceeding the endoprosthetic device’s functional life [2].

TFR is considered to be the one of the most demanding surgical techniques. It includes the use of modular megaprosthesis that replaces both the hip joint and the knee joint [3]. In addition, TFR is often performed as a revision arthroplasty in patients who have already undergone multiple operations in the area. Those patients often have massive bone loss, poor bone quality and less compliant soft tissues [4]. These patient factors and the surgical technique are why TFR is associated with high complication rates [5]. The literature describes various complications including periprosthetic infection, mechanical failure, dislocations and others, often leading to revision surgeries or even amputation [4]. Some authors have used the Henderson classification to divide the complications into five categories [6]. However, the literature lacks systematized complication reports with described management and prevention.

For these reasons, we decided to write this article to present options for management of complications after the TFR procedure. We aimed to answer the following questions: 1. What were the most common complications in our experience? 2. How to can they be handled? 3. What could be done to minimize the risk of complications?

## 2. Methods

In this study a series of total femoral replacements from two independent hospitals, both functioning as tertiary referral centers, was retrospectively reviewed. Eligible patients were identified from the hospital database. The inclusion criteria were TFR after tumor resection or mechanical failure of THR and/or TKR accompanied or not by periprosthetic or interprosthetic fracture. Patients with follow-up shorter than 6 months were excluded.

Patient charts were retrospectively reviewed and the initial reconstruction (THR or/and TKR), number and type of revision surgeries prior to TFR, date of TFR, indication for TFR, patients’ age at TFR procedure, type of TFR implant, TFR procedure description, revision surgeries after TFR with descriptions, any reports of complications and their treatment were recorded. The main characteristics were collected in a spreadsheet in MS Excel. Preoperative, postoperative, and follow-up radiographs were reviewed and compared with clinic notes and operative reports. Due to the number of patients, the analysis of results was limited to descriptive methods.

The procedures were performed in accordance with the ethical standards of the Institutional Scientific Committee as well as with the 1964 Helsinki Declaration and its later amendments. We obtained an IRB approval number, AKBE/59/13. Due to the retrospective nature of the study, the need to obtain informed consent was waived.

## 3. Results

A total of 19 patients who met the inclusion criteria were treated with a TFR between 2011 and 2023. There were 15 women and 4 men; the mean age at the time of the TFR procedure was 67 years (standard deviation: ±10.46; range: 49 to 81 years). In 11 cases the primary diagnosis was degenerative joint disease of the hip and/or knee for which the patients were treated with THR and/or TKR. All non-oncologic patients were operated on in one hospital by the same surgeon except for one case where intramedullary TFR was utilized—this patient was treated in the clinic where oncologic TFR was performed. Ten of these patients underwent revision surgeries of THR and/or TKR prior to TFR; the number of revisions varied from one to four. There were eight cases of metastatic tumors as primary diagnosis. All oncologic cases were handled in one hospital by the same surgeon. In four cases TFR was the first reconstruction following tumor resection and in four patients it was used as a revision endoprosthesis after local progression of metastasis in patients who already had proximal femoral replacement (PFR).

**Figure 1:**
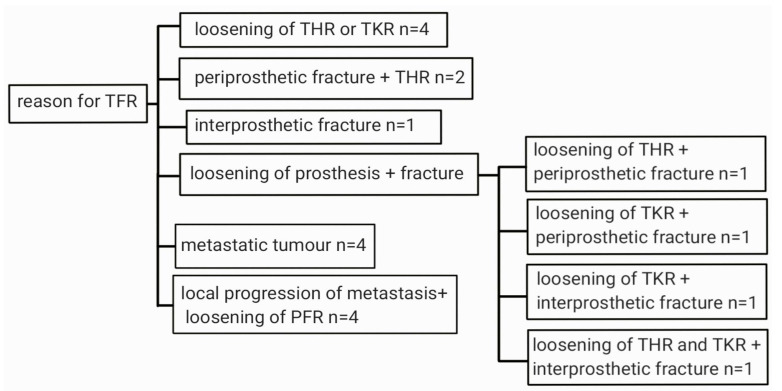
Flow chart showing indications for total femur replacement (TFR) (TKR: total knee replacement; THR: total hip replacement; PFR: proximal femoral replacement).

Indications for the THR were metastatic tumors, local progression of metastasis with loosening of PFR, loosening of THR or TKR, periprosthetic fractures and interprosthetic fractures often accompanied by poor bone quality (Figure 1). The following TFR implants were used: 12 Mutars (Implantcast), two Orthopaedics Salvage Systems (Zimmer-Biomet), one Megasystem (LINK), one Limb Preservation System (DePuy Synthes), three Global Modular Replacement Systems (Stryker) and one Mutars (Implantcast) Intramedullary Total Femoral Replacement. There were no hip hemiarthroplasties included in this study. The acetabular component had a standard bearing in 11 cases and a dual mobility bearing in eight cases.

Ten patients (53%) suffered local complications. Hip dislocations were reported in four (21%) individuals, two of whom were reduced without surgery and treated conservatively. Two patients needed a surgical reduction with either acetabulum or head and inlay replacement. A mechanical loosening of the acetabulum was observed in one case and resulted in a revision surgery with a SPACER implantation later replaced with a custom-made acetabular implant. One patient had a conflict between the stem and a custom-made acetabular implant which was resolved by a revision surgery with changing of the head and proximal femur.

Infection was one of the most common complications; it occurred in four patients (21%). In two cases, revision surgery with debridement, application of a layer of cement with antibiotics around the prosthesis, and Stimulan application (vancomycin) were needed. Wound healing problems were noticed in five patients (26%), with one case of necrosis, and were treated with surgical wound cleaning, VAC therapy and antibiotic therapy when accompanied by infection. Necrosis was treated firstly with surgical wound cleaning and VAC therapy and later a local skin flap was added. One patient (5%) presented symptoms of an allergic reaction and had positive tests for chrome and nickel; as the allergy was worsening with time, the patient was scheduled for a revision surgery to replace the prosthesis with hypoallergenic-coated one.

Six patients (32%) underwent revision surgeries after the TFR procedure. The number of revisions varied from one to four and managed patient-specific complications as described in Table 1. No patients required amputation.

## 4. Discussion

The study summarizes 19 cases of TFR performed in oncological and revision patients in two orthopedic clinics. In 11 cases the primary diagnosis was degenerative joint disease of the hip and/or the knee, while in eight cases the primary diagnosis was oncological. The most common indications to perform TFR in non-oncologic patients were ex aequo loosening of THR/TKR (*n* = 4) and loosening of THR/TKR with concomitant fracture (*n* = 4), while in oncologic patients it was local progression of metastasis with loosening of PFR (*n* = 4) and metastatic tumors (*n* = 4). In this study there were no patients with primary bone tumors, which can be an indication for TFR. We put an emphasis on the complications that arose during the therapeutic process with the goal to share our experience and allow the readers to avoid such situations. The most common complications in our study were wound healing problems (*n* = 5), infection (*n* = 4), and dislocations (*n* = 4).

Patients undergoing TFR can be divided into two main groups based on the primary diagnosis prior to the procedure: oncologic and non-oncologic patients. To investigate the differences between the groups, the authors design studies with only oncologic patients [7], only non-oncologic patients [4], and studies including patients from both groups [6]. In the systematic review of TFR, the authors present that oncologic patients have higher rates of soft tissue failures, aseptic loosening, and structural failures. On the other hand, the study shows that non-oncologic patients have a higher rate of prosthetic infections and worse functional outcomes [8]. In this study, both oncologic and non-oncologic patients were included. We observed fewer complications and fewer revisions after TFR in oncological patients. Our hypothesis is that this may be caused by a lower number of revisions prior to TFR in oncologic patients. Nevertheless, the observations have to be interpreted cautiously, taking into account the small sample size in the study.

The most frequent complication of TFR described in the literature is periprosthetic infection [8]. The incidence of this complication varies from 10 to 50% depending on the author [5,9]. In our study 21% of the patients suffered periprosthetic infection, which seems to concur with the results previously described in the literature [10,11]. This consistency suggests that our findings align with previously reported complication ranges for megaprosthetic and limb salvage procedures, although direct comparisons remain limited due to differences in study design and patient populations. Management of the infection with total femur prosthesis requires careful consideration of the treatment plan in every case. Hardware removal is not always the best option since inserting a new revision implant would mean further bone destruction. In the management of the infected TFR patients, we utilized antibiotic therapy, surgical debridement, cement with antibiotics and implant removal in different combinations. We did not observe the superiority of any method above others. Further studies focusing on optimal infection management after TFR should be considered to establish evidence-based recommendations resolving this issue.

The management of wound healing problems after TFR remains a serious clinical challenge. The extensive surgical approach, in many cases combined with a history of multiple revisions, makes the wound after TFR incomparable with wounds after primary joint replacement, especially those utilizing minimally invasive approaches such as direct anterior approach (DAA) in THR [12]. The surgical approaches utilized in TFR include one anterolateral incision, proximal anterolateral incision with distal midline incision, and in some cases a parapatellar approach [13]. Each patient requires individual planning considering the scars after previous operations. When wound healing complications occur, solutions such as VAC therapy, dressings containing silver ions and even surgical debridement should be considered.

Recurrent dislocations are well described in patients with TFR [4,7,14]. In our study the dislocation rate reached 21%. In two cases the dislocations were managed by repeated closed reduction, and in two cases open reduction with either head and inlay exchange or acetabulum replacement was necessary. In our experience the way to manage this complication should be identified considering the full clinical view and the patient’s condition. In some patients dislocations occur repeatedly, causing a limitation in the patient’s mobility, while in others dislocation can be a single event resolved by closed reduction. The possibility of using dual-mobility cups should be considered as a way to decrease the percentage of patients suffering from TFR dislocations [10].

Allergic reaction caused by the implant is a rare complication described both in THR and TKR cases [15]. One of the patients included in this study had an allergic reaction to their TFR implant (Mutars (Implantcast)). In this case the patient had positive tests for chrome and nickel allergy. Although this type of complication occurs rarely, the possibility of an allergic reaction cannot be neglected and an adequate treatment plan, including revision surgery with implant exchange, should be prepared.

The case of skin necrosis described in this study occurred in the patient where an extended approach to the femur had to be utilized. The patient had a THR, followed by a periprosthetic fracture with internal fixation using a plate and screws, as well as two other revision surgeries in the region, which may have caused extensive adhesions and angiogenesis. This type of complication is described in THA and TKA [16,17]; however, the occurrence in TFR should not be a surprise since the procedure often requires extension of the surgical approach and is frequently performed after several revision surgeries in the area.

TFR is a procedure that requires substantial bone extraction that affects postoperative function of the operated limb. Removing the entire femur causes problems with the muscles that attach to this bone. However, in some cases, the solution is able to provide relief to the soft tissue around the femur. In a non-oncologic patient with interprosthetic fracture and osteoporosis, we applied Mutars intramedullary TFR. This type of implant allows one to spare the midsection of the femoral shaft and should be considered in patients where there is no need for entire femur removal.

### Limitations

The results of the study cannot be interpreted without carefully considering its limitations. First, our study had a relatively small sample size. However, due to the rarity and complexity of the procedure it is not uncommon to publish studies with study groups smaller than 20 patients [9,11,18,19]. Nevertheless the results should be interpreted with caution. We believe that the results add to the literature, showing the experience of clinics developing their TFR program in oncological and non-oncological patients and the challenges they are faced with. Second, the patient data was retrospectively gathered. A prospective study on TFR should be taken into account. Next, the follow-up period of the individual patients who were included in the study differs. This could have affected the long-term complications observed. An important limitation was that the patient documentation was not always complete due to prolonged treatment in different clinics. That may have affected the amount of information collected about diagnosis, revisions, and other treatment prior to TFR. What is more, our study could not summarize some factors that may have affected patients’ results due to missing data, such as American Society of Anesthesiologists score or the type of physical therapy that was utilized [20]. Additionally, the variability in indications for surgery and patient characteristics introduces important sources of heterogeneity that may influence the outcomes. Finally, our study lacks standardized outcome measures. Due to the retrospective data collection we were not able to include functional measurement tools in our study.

## 5. Conclusions

These findings suggest that total femur replacement remains a high-risk but viable limb salvage option after failed arthroplasty or in cases of bone tumors. The outcomes seem to be closely influenced by the complication profile and individualized management strategies. In our experience, the most frequent complications are local infections and wound healing problems. Effective treatment of deep infection should include antibiotic therapy, surgical debridement, and the use of antibiotic-loaded cement to cover the prosthesis. In severe cases, a two-stage revision with partial removal of the implant and reimplantation after the infection has resolved may be necessary. We would like to point out the importance of salvage of the soft tissues for the best stability and function. Based on our experience, we propose ways of minimizing complications: in non-oncological patients, consider TFR at an earlier stage—before patients undergo many revision surgeries—especially in elderly patients; take into consideration the possibility of metal allergy; consider performing an angio-CT before the surgery; plan the implant length based on the length of the femur in the contralateral limb; during dissection, ensure the continuity of the distal attachment of the gluteus medius muscle and the proximal attachment of the vastus lateralis muscle; use the special holes in the prosthesis body to reconstruct the natural attachments for these muscles during suturing; use dual-mobility bearing to decrease the occurrence of dislocation; use local antibiotics (e.g., Stimulan) during the procedure in cases where TFR is used as a revision system; apply VAC therapy after the procedure to avoid wound healing problems; and, after the surgery, consider using an anti-dislocation brace.

These recommendations may support clinical decision-making but should always be adapted to individual patient characteristics and should be interpreted as experience-based insights derived from our cohort rather than definitive or generalizable treatment guidelines.

## Figures and Tables

**Table 1:** Table describing each patient’s characteristics. Initial reconstruction, number of revision surgeries prior to TFR, reason for TFR, type of implant, complications that occurred with management and number of surgical interventions post TFR.

Patient Number	Initial Reconstruction	Number of Revisions Prior to TFR	Indication for TFR	Type of TFR Implant	Complications	Management of Complications	Number of Revision Surgeries Post TFR
1	-	0	metastasis of renal cancer	Stryker (GMRS) with dual mobility bearing	-		0
2	-	0	metastasis of breast cancer	Stryker (GMRS) with dual mobility bearing	-		0
3	-	0	metastasis of breast cancer	Stryker (GMRS) with dual mobility bearing	-		0
4	-	0	metastasis of breast cancer	Mutars (Implantcast)	-		0
5	PFR Mutars	0	local progression of metastasis with loosening of PFR	Mutars (Implantcast)	-		0
6	PFR Mutars	0	local progression of metastasis with loosening of PFR	Mutars (Implantcast)	-		0
7	PFR Mutars	0	local progression of metastasis with loosening of PFR	Mutars (Implantcast)	wound healing problems with discharge	antibiotic therapy	0
8	PFR Mutars	0	local progression of metastasis with loosening of PFR	Mutars (Implantcast)	dislocation	revision surgery: open reduction with acetabulum replacement	1
9	TKR and PFR	0	interprosthetic fracture + osteoporosis	Mutars Intramedullary TFR with dual mobility bearing	-		0
10	THR	1	loosening of THR + poor bone quality	LPS (Synthes)	recurrent dislocations	closed reduction of the dislocation	0
11	THR and TKR	2	interprosthetic fracture + loosening of THR and TKR	Mutars (Implantcast)	-		0
12	THR	1	loosening of THR	Mutars (Implantcast) with dual mobility bearing	dislocation	revision surgery: open reduction and a head and inlay replacement	1
13	THR and TKR	1	interprosthetic fracture	OSS (Zimmer-Biomet)	infection with discharge leading to a fistula; wound healing problems	antibiotic therapy; first revision surgery: debridement with surgical wound cleaning, application of a layer of cement with antibiotics around the prosthesis and Stimulan application (vancomycin); second revision surgery: removal of the head, debridement with surgical wound cleaning and removal of the fistula, Stimulan application (vancomycin)	2
14	THR	3	loosening of THR	Mutars (Implantcast) with dual mobility bearing	infection with purulent discharge; mechanical loosening of the acetabulum	first revision surgery: removal of the acetabulum and head leaving the patient with a spacer; second revision surgery: removal of the spacer and proximal femur and Stimulan application (vancomycin); third revision surgery: debridement, application of a layer of cement with antibiotics around the prosthesis and Stimulan application (vancomycin); fourth revision surgery: placement of a custom made acetabulum and attachment of the proximal femur	4
15	THR and TKR	2	interprosthetic fracture + loosening of TKR	OSS (Zimmer-Biomet)		Stimulan application during TFR surgery (vancomycin and gentamicin) as prophylaxis	0
16	THR	3	periprosthetic fracture + loosening of THR	Mutars (Implantcast)	wound healing problems leading to necrosis	surgical cleaning of the wound and VAC therapy	1
17	THR	2	loosening of THR with osteolysis	Mutars (Implantcast) with dual mobility bearing	infection leading to purulent discharge; wound healing problems; conflicting stem; acetabular custom-made implant	VAC therapy and antibiotic therapy; first revision surgery: patellar prosthesis; second revision surgery: replacement of the head and proximal femur due to the stem- acetabulum conflict; third revision surgery: replacement of the prosthesis for a hypoallergenic coated implant	3
18	THR	4	periprosthetic fracture	Mutars (Implantcast) with dual mobility bearing	infection leading to purulent discharge; wound healing problems	surgical cleaning of the wound; VAC therapy; antibiotic therapy	1
19	TKR	2	periprosthetic fracture + loosening of TKR	Megasystem (LINK)	recurrent dislocations	repeated closed reduction of the dislocation	0

TFR: total femur replacement; TKR: total knee replacement; THR: total hip replacement; PFR: proximal femoral replacement.

## Data Availability

The original contributions presented in this study are included in the article. Further inquiries can be directed to the corresponding author.
